# Injury of the posterolateral corner of the knee does not lead to higher intra‐articular external tibial rotation in the MRI

**DOI:** 10.1002/jeo2.70360

**Published:** 2025-07-13

**Authors:** Jana Sobota, Christian Arras, Jannik Frings, Milan Sobota, Karl‐Heinz Frosch, Matthias Krause

**Affiliations:** ^1^ Department of Trauma and Orthopedic Surgery University Medical Center Hamburg‐Eppendorf Hamburg Germany; ^2^ Sir William Dunn School of Pathology University of Oxford Oxford UK; ^3^ Department of Trauma Surgery, Orthopaedics and Sportstraumatology BG Klinikum Hamburg Hamburg Germany

**Keywords:** dial test, knee, MRI, posterolateral corner, tibial rotation

## Abstract

**Purpose:**

The aim of this study was to evaluate external tibial rotation using magnetic resonance imaging (MRI) in patients with injuries to the posterolateral corner (PLC) of the knee.

**Methods:**

A retrospective analysis of 26 patients with PLC injury (study group) was performed and compared with a control group of 100 patients without knee injuries. Intra‐articular tibial rotation was assessed in MRI measuring two angles: (1) between the surgical epicondylar axis (SEA) of the femur and the posterior tibial condyle line (PTC) (SEA‐PTC); and (2) between the posterior femoral condyle (PFC) and the PTC (PFC‐PTC). Results were aligned with the dial test (DT), representing the clinical assessment of PLC injuries. The statistical significance of differences in SEA‐PTC and PFC‐PTC was evaluated using a two‐sided Welch's *t* test.

**Results:**

The mean SEA‐PTC was −6.3 ± 7.9° (study group) versus −6.1° ± 4.0° (control group), and the mean PFC‐PTC was −5.2 ± 2.4° (study group) versus −5.4 ± 1.5° (control group). There were no statistical differences regarding the SEA‐PTC or the PFC‐PTC between both groups (*p* = 0.87 and *p* = 0.87, respectively). DT was positive in 80% of patients with PLC injury; thus, sensitivity was 80%. SEA‐PTC and PFC‐PTC were not statistically different between DT positive/negative patients (*p* = 0.89 and *p* = 0.1, respectively). There was a significant correlation between SEA‐PTC and PFC‐PTC (*R* = 0.88; *p* < 0.001).

**Conclusion:**

PLC injuries are not associated with increased external tibial rotation in the MRI, while the DT is a valuable method for the clinical assessment of PLC injuries.

**Level of Evidence:**

Level III.

**Abbreviations**
AC: arcuate complex
DT: dial test
LCL: lateral collateral ligament
MRI: magnetic resonance imaging
PCL: posterior cruciate ligament
PFC: posterior femoral condyle
PFL: popliteofibular ligament
PLC: posterolateral corner
PM: popliteus muscle
PT: popliteus tendon
PTC: posterior tibial condyle
SEA: surgical epicondylar axis

## INTRODUCTION

Injuries of the posterolateral corner (PLC) of the knee are difficult to detect [16]. However, they constitute up to 16% of knee ligament injuries [16]. If untreated, they can result in complex knee instability and development of osteoarthritis [4, 5].

The main defining structures of the PLC are the popliteus muscle (PM) and popliteus tendon (PT), the popliteofibular ligament (PFL) and the lateral collateral ligament (LCL) [4, 11]. The PFL is part of the arcuate complex (AC), which is a group of fibres attached to the PT, the thickest of which is the PFL [17]. However, they are of lesser importance regarding the stability of the knee [11]. Injuries of the PLC are frequently accompanied by rupture of the posterior cruciate ligament (PCL) or less frequently by injuries of the anterior cruciate ligament (ACL) [2, 10, 14].

The PLC stabilizes the knee against varus forces and external tibial rotation [14]. The main stabilizers against excessive external tibial rotation are the PT and PFL [2]. The LCL is the main stabilizer against varus stress, but it also prevents external tibial rotation [14].

Diagnosis of PLC injuries can be difficult. Clinical tests to assess possible PLC injury include the varus stress test, the posterolateral drawer test and the dial test (DT) [4]. The DT is considered effective in diagnosing PLC injury [1]; however, studies investigating the sensitivity or specificity of this test are lacking [4, 9, 18].

Magnetic resonance imaging (MRI) is usually performed to assess knee pathologies, especially ligamentous injuries [4]. Thorough knowledge of the radiological appearance, both normal and abnormal, of the key structures of the PLC is crucial to detect PLC injuries, especially in clinically ambiguous cases [15]. In MRI, injury to the structures of the PLC may present as oedema surrounding the tendon or as tendon rupture. Indirect signs may include knee joint effusion, although this is not specific to PLC injury [11]. Furthermore, Sensöz et al. have described the medial femoral notching sign as an indicator of PLC injury [13]. Nevertheless, identifying PLC injuries on MRI scans can still be difficult, especially in chronic cases [11]. Therefore, additional diagnostic tools might be beneficial in avoiding missed diagnoses of PLC injury.

Farinelli et al. assessed the impact of injury of the anterolateral corner (ALC) of the knee on intraarticular tibial rotation, whereby injuries were associated with increased internal rotation in MRI [6]. In their study, the angle between the surgical epicondylar axis (SEA) of the femur and the posterior tibial condyle (PTC) line of the tibia was measured (SEA‐PTC). The results were compared between patients with ALC injuries and those without. Patients with ALC injury had a greater SEA‐PTC angle, hence the internal tibial rotation was increased [6].

Concerning PLC injuries, there are no diagnostic tools for the assessment in MRI scans. We hypothesize that as the PLC stabilizes the knee against external tibial rotation, injury to the PLC results in increased external tibial rotation.

## MATERIALS AND METHODS

In this retrospective study, 26 patients with PLC injuries who presented to our clinic between 2019 and 2024 were analyzed and compared to 100 ‘knee‐healthy’ controls without any clinically or radiographically apparent ligamentous knee injury.

PLC injury was considered present if at least one of the following structures was injured based on MRI findings: PT, PM, PFL and LCL. Acute and chronic injuries were included. Injuries were considered acute, when they had happened ≤30 days prior to MRI imaging. Injuries ≥31 days were considered chronic. Moreover, patients who received conservative as well as surgical treatment were included. Patients with an intact PLC in MRI were excluded.

MRI examinations were performed with the patient in the supine position and ideally with the knee in full extension. In cases of recent injury with subsequent effusion where full extension was not possible, a slight bending of the knee was allowed with flexion up to 15°.

**Figure 1:**
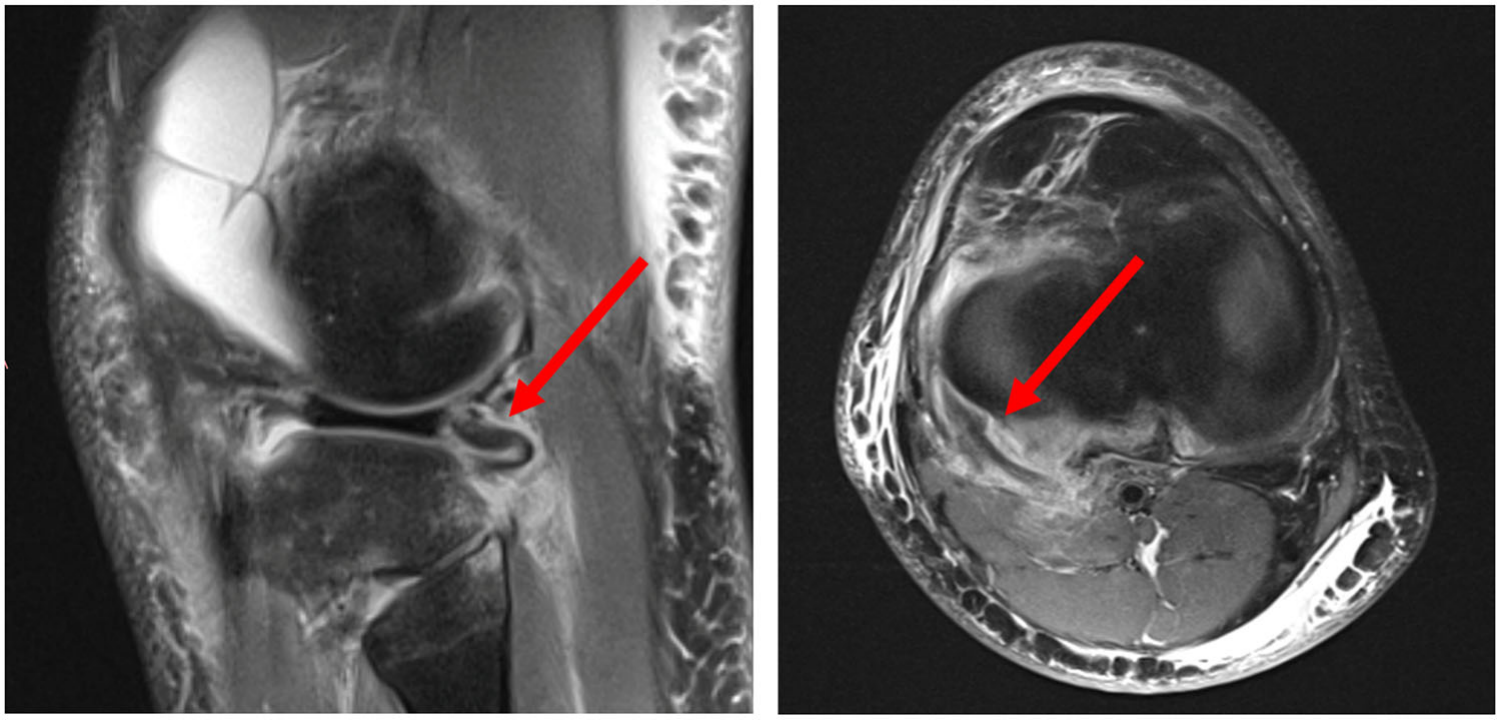
Sagittal (left) and axial (right) MRI scan with a ruptured popliteus tendon (red arrow). MRI, magnetic resonance imaging.

Sagittal, axial and coronal MRI planes were thoroughly assessed for injuries of the PT, PM, PFL, LCL and PCL. Injury was considered present if there was oedema in the tendon or muscle, or if the structure was (partially or completely) ruptured. Diagnosis of PLC injury was confirmed if at least one of these structures was injured (Figure 1). As PLC injury is often accompanied by PCL rupture, the latter was also assessed. PCL injury was considered present if the ligament was partially or completely ruptured.

### External tibial rotation

**Figure 2:**
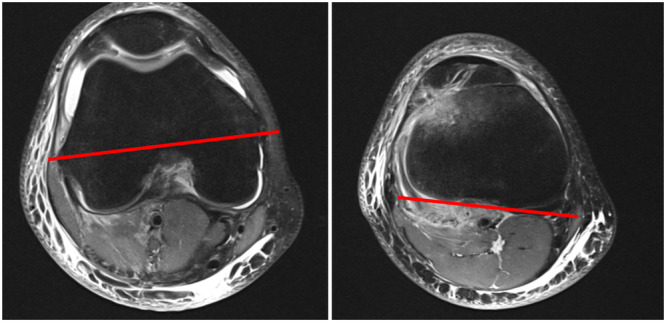
Measurement of SEA‐PTC angle. The SEA is extending from the tip of the lateral epicondyle to the medial sulcus located just below the medial epicondyle and the PTC extends from the medial to lateral tibial condyle. PTC, posterior tibial condyle; SEA, surgical epicondylar axis.

**Figure 3:**
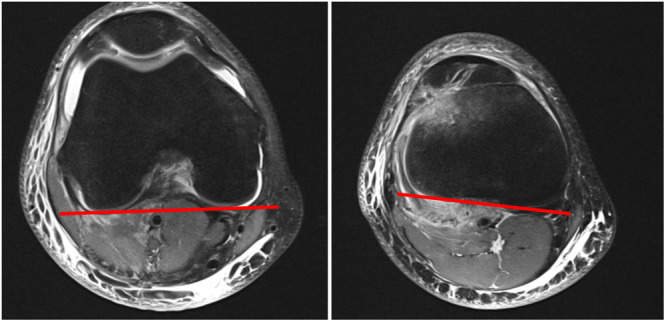
Measurement of PFC‐PTC angle. The PFC is extending from the tip of the lateral to the medial posterior femoral condyle. The PTC extends from the medial to lateral posterior tibial condyle. PFC, posterior femoral condyle; PTC, posterior tibial condyle.

Two angles were measured in axial MRI scans:

1. SEA‐PTC: The angle between the SEA of the femur and the PTC line (Figure 2) according to Farinelli et al. [6]. Internal rotation was documented as negative values and external rotation as positive values.
2. PFC‐PTC: The angle between the tangent lines to the posterior femoral condyle (PFC) and the PTC was measured as described by Huettner et al. [8]. The PFC‐PTC angle was termed as knee version [8] (Figure 3). This angle was also measured in both the study and the control group.

SEA‐PTC and PFC‐PTC angles were to be compared between patients with and without injury of the PLC. Furthermore, results were to be aligned with the clinical assessment of PLC injuries.

### Clinical examination

A standardized and systematic clinical examination was conducted on all knees. For the clinical assessment of PLC injuries, the DT was performed in prone position, with application of external tibial rotation in 30° and 90° flexion of the knee. Increased rotation in 30° flexion compared to the contralateral side was documented as a positive DT. Results of the DT were documented as binary data—either DT positive or DT negative.

### Statistical analysis

All values are reported as mean/median values ± SDs for descriptive analysis. Statistical analysis was performed using R Studio software version 4.4.1. The statistical power of the study was assessed to ensure an adequate sample size. Post hoc power analysis revealed that the study had a power of 80% for SEA‐PTC and a power of 70% for PFC‐PTC, calculated based on an expected effect size of 2.5° in external tibial rotation. The statistical significance of differences in SEA‐PTC and PFC‐PTC between the study and control groups was tested using a two‐sided Welch's *t* test. *p* Values less than 0.05 were considered statistically significant. A linear regression was performed to assess the correlation of SEA‐PTC and PFC‐PTC after calculating the mean values of SEA‐PTC and PFC‐PTC for each group.

## RESULTS

Twenty‐six patients with PLC injury were included in the study. Of the 26 patients with PLC injury (study group), 65% (*n* = 17) were male and 35% (*n* = 9) were female. The median age of the patients was 34 ± 16 years (range: 17–64). In the control group of 100 ‘knee healthy’ patients, 52% (*n* = 52) were male and 48% (*n* = 48) were female. The median age of the control group was 26 ± 4 years (range: 20–50).

In the study group, PLC injuries (PM/PT, PFL and LCL) were evaluated. Seventy‐eight per cent (*n* = 20) of injuries were acute, whereas 22% were chronic. Within the study group, 80% (*n* = 21) of patients received surgical treatment, whereas five patients were treated with non‐operative measures such as physiotherapy. The most frequent injury was to the PM/PT (88%, *n* = 23). Injury of the LCL and PFL was roughly equally distributed: 50% (*n* = 13) had LCL injury and 54% (*n* = 14) had an injury of the PFL. Eighty‐five per cent (*n* = 22) of patients with PLC injury had concomitant injury of the PCL (Table 1).

**Table 1:** Distribution of injury of the PLC and PCL in the study cohort (*n* = 26).

Injured structure	*n*	%	Combined injury	*n*	%
PM/PT	23	88	PM/PT + PFL	13	50
PFL	14	54	PM/PT + PCL	19	73
LCL	13	50	PM/PT + LCL	12	46
			PFL + LCL	7	27
PCL	22	85	PFL + PCL	12	46
			PCL + LCL	12	46
			PT + LCL + PFL	7	27
			PT + LCL + PFL + PCL	6	23

Abbreviations: LCL, lateral collateral ligament; PCL, posterior cruciate ligament; PFL, popliteofibular ligament; PLC, posterolateral corner; PM, popliteus muscle; PT, popliteus tendon.

### External tibial rotation

SEA‐PTC and PFC‐PTC were measured in all PLC injured patients (*n* = 26) and in the control group (*n* = 100). In the study group, the mean SEA‐PTC was −6.3 ± 7.9° (range: −20.4 to 9.9) and −6.1 ± 4.0° (range: −19.9 to 4.0) in the control group. Mean PFC‐PTC was −5.2 ± 2.4° (range: −15.6 to 9.0) in the study group and −5.4 ± 1.5° (range: −17.0 to 4.0) in the control group. SEA‐PTC and PFC‐PTC were not statistically different between the study and control groups (Table 2).

**Table 2:** SEA‐PTC and PFC‐PTC angles (degrees) in PLC injured patients (study) and control group.

	Study group	Control group	*p*
SEA‐PTC (degrees)			
Mean	−6.3	−6.1	*ns*
Median	−6.6	−6.0	
SD	±7.9	±5.8	
Min	−20.4	−19.9	
Max	9.9	4.0	
PFC‐PTC (degrees)			
Mean	−5.2	−5.4	*ns*
Median	−5.9	−5.4	
SD	±2.4	±1.5	
Min	−15.6	−17	
Max	9.0	4.0	

Abbreviations: PFC, posterior femoral condyle; PLC, posterolateral corner; PTC, posterior tibial condyle; SD, standard deviation; SEA, surgical epicondylar axis.

Angles were compared between patients with LCL injury and those with intact LCL, but no significant differences were found between the two groups. Additionally, SEA‐PTC and PFC‐PTC were compared between patients with acute and chronic injuries, and the results revealed no statistically significant differences.

**Figure 4:**
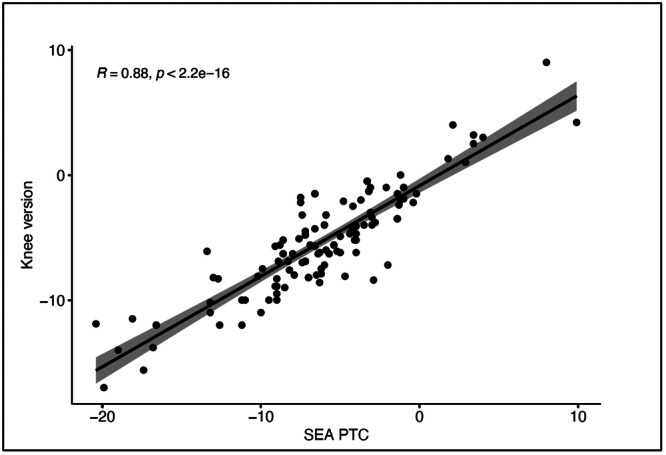
Correlation between SEA‐PTC and knee version (PFC‐PTC) for the total cohort. Data points are jittered to avoid overplotting. PFC, posterior femoral condyle; PTC, posterior tibial condyle; SEA, surgical epicondylar axis.

Linear regression was performed to assess the correlation of SEA‐PTC and PFC‐PTC. There was a significant correlation between the SEA‐PTC and PFC‐PTH (*r* = 0.88, *p* < 0.001) for the total cohort (Figure 4). When stratifying the data by whether PLCs were injured (study group) or not (control group), there was also a significant correlation (*r* = 0.76; *p* < 0.001 and *r* = 0.79; *p* < 0.001, respectively). The high correlation indicates that both angles consistently measure external tibial rotation.

### Clinical examination—DT

The DT was assessed in 25 patients with PLC injury (96%). The test was true positive in 20 patients and false negative in 5. The sensitivity of the DT was 80% (20/25). External tibial rotation was also compared between patients with a positive (*n* = 20) and a negative (*n* = 5) DT. There was no statistical difference between the angles between the study and control groups; however, the assessed number of patients is very small (Table 3).

**Table 3:** SEA‐PTC and PFC‐PTC angles (degrees) in PLC injured patients (study group) with positive and negative dial test (DT).

	DT positive (*n* = 20)	DT negative (*n* = 5)	*p*
SEA‐PTC (degrees)			
Mean	−6.1	−5.8	*0.887*
Median	−6.6	−5.7	
SD	±7.9	±2.5	
Min	−20.4	−8.8	
Max	9.9	−2.9	
PFC‐PTC (degrees)			
Mean	−4.6	−7.2	*0.1014*
Median	−5.5	−7.5	
SD	±5.8	±1.5	
Min	−15.6	−8.6	
Max	9.0	−5.6	

Abbreviations: PFC, posterior femoral condyle; PLC, posterolateral corner; PTC, posterior tibial condyle; SD, standard deviation; SEA, surgical epicondylar axis.

## DISCUSSION

Detection of injuries of the PLC is important for optimal treatment of knee injuries, as they often accompany other injuries, such as rupture of the ACL or PCL [10, 11]. The structures of the PLC of the knee are proven to be preventing increased external tibial rotation [5, 14]. The aim of this study was to address whether injury of the PLC leads to MRI‐detectable increased intra‐articular tibial external rotation. This hypothesis was not confirmed in this study, as we did not find increased external rotation in knees with PLC injury in MRI scans.

First, it could be assumed that externally applied force seems to be necessary to prompt increased external tibial rotation in PLC injured knees. Domnick et al. conducted a human cadaveric study in which they tested the stabilizing effects of the different structures of the PLC, whereby 5 N m external rotatory loads were applied to the cadaveric knee [5]. A significant increase in external rotation was found in knees with injury of the PLC, especially in those with rupture of the PFL [5]. Moreover, in another biomechanical study by Strauss et al., the effect of anterior force when testing external rotation in PLC‐deficient knees was examined [14]. They found an increased external tibial rotation if an additional anterior force was applied. Thus, the combination of externally applied force as well as an anterior counterfort, as it is applied during the DT in prone position, seems important in prompting increased external tibial rotation in PLC‐deficient knees [14]. As the patient is examined in MRI in a lying supine position with a relaxed knee, this force has been lacking to evoke external tibial rotation. This might explain the absence of increased external tibial rotation in the assessed MRI scans.

This theory is consistent with the findings of the DT, which showed that 80% of patients with a positive DT had an injury to the PLC. During the DT, a rotational force is applied to the tibia, ideally while the patient is in a prone position with an anterior counterforce. A positive DT is indicated by increased external tibial rotation compared to the uninjured contralateral side. Therefore, applying forced external tibial rotation along with an anterior counterforce appears essential for eliciting increased external tibial rotation in knees with PLC injuries. In contrast, when patients undergo MRI in a supine position with a relaxed knee, there is no anterior counterforce or external rotational force applied, which may account for the absence of detectable external tibial rotation in the MRI scans. This explanation is supported by the statistical indifference of SEA‐PTC and PFC‐PTC values between patients with a clinically proven positive or negative dial test. Consequently, the lack of stability resulting in increased external tibial rotation due to PLC injury is not apparent in static MRI, despite being identifiable through clinical assessments using the dial test. Therefore, the measurement of SEA‐PTC and PFC‐PTH might have greater diagnostic accuracy in dynamic rather than static MRI. However, dynamic MRI is not widely used in clinical practice [7]. In the literature, studies on the accuracy of the DT are lacking. Yet, a human cadaveric study by Bae et al. showed that the DT is a valuable diagnostic tool in detecting PLC injuries [1]. In our study, we found a sensitivity of the DT of 80%. This underlines the importance of clinical examination of the knee.

Bonadio et al. compared the results of MRI of the PLC with the clinical assessment of the PLC under anaesthesia [3]. They found MRI to be less sensitive than the clinical examination. In their study, the time span between knee trauma and MRI was comparable to the time span in our study. They concluded, that especially chronic cases of PLC injury are difficult to detect in MRI, and enhanced the importance of clinical examination [3]. Rakhra et. al. found a higher sensitivity of MRI for detection of PLC injuries, however in their study acute cases with a mean duration within injury and MRI of 4 days were assessed. Moreover, in their study of acute cases of PLC injuries, they found that MRI results aligned well with intraoperative surgical findings. Nevertheless, Rakhra et al. still underlined the importance of the clinical examination [12].

Another reason for the lack of increased external rotation might be the positioning of the patient during the MRI of the knee: The patient is lying on the back with the knee fully extended or in slight flexion. The examined leg is positioned in a mount which keeps the knee still to avoid artefacts in the scans. First, this mount might prevent the tibia from rotating externally. More importantly, the extension of the leg might hinder external tibial rotation. In the aforementioned study by Domnick et al., increased external tibial rotation was found in 60° and 90° flexion of the knee [5]. Therefore, the extent of flexion seems to play a role in the extent of external rotation. If intra‐articular rotation had been assessed at greater knee flexion, we might have observed increased external tibial rotation in the injured knees.

There are some limitations to this study. First, the cohort of PLC injured patients is relatively small, primarily because such injuries are infrequent. Although retrospective power analysis confirmed that the study group size was adequate, additional studies with a larger patient population would be beneficial to validate the findings and enhance their reliability. Additionally, tibial rotation measurements were not compared to the contralateral side, meaning individual rotation deviations were not accounted for. Furthermore, factors such as femoral or tibial torsion and femoral anteversion were not considered. The alignment of the MRIs may have also impacted the measurements, as any imperfections in reconstructing the MRI slices could lead to discrepancies in the results.

## CONCLUSION

Injury of the PLC of the knee cannot reliably be detected in MRI by measuring the intra‐articular rotation of the tibia. There are no differences in intra‐articular external tibial rotation in PLC injured and uninjured knees. External forces need to be applied to prompt increased external rotation in PLC injured knees, as performed in the DT. Comprehensive clinical examination and thorough MRI evaluation remain necessary for diagnosing PLC injury.

## AUTHOR CONTRIBUTIONS

*Conceptualization:* Jana Sobota, Matthias Krause and Karl‐Heinz Frosch. Methodology: Jana Sobota, Matthias Krause and Milan Sobota. *Formal analysis and investigation:* Jana Sobota, Christian Arras, Matthias Krause, Jannik Frings and Milan Sobota. *Writing—original draft preparation:* Jana Sobota and Matthias Krause. *Writing—review and editing:* All authors. *Resources:* Jana Sobota, Matthias Krause, Jannik Frings and Karl‐Heinz Frosch. *Supervision:* Matthias Krause and Karl‐Heinz Frosch. *Project administration:* Matthias Krause.

## CONFLICT OF INTEREST STATEMENT

The authors declare no conflicts of interest.

## ETHICS STATEMENT

This study was approved by the Clinical Ethics Committee of University Medical Center Hamburg‐Eppendorf, and ethical approval was obtained under protocol number (2024‐101279‐BO‐ff). Informed consent was obtained from all participants in the study.

## Data Availability

Research data are not shared.
